# Pediatric chronic nonbacterial osteomyelitis of the mandible: Seattle Children’s hospital 22-patient experience

**DOI:** 10.1186/s12969-019-0384-8

**Published:** 2020-01-15

**Authors:** Austin Gaal, Matthew L. Basiaga, Yongdong Zhao, Mark Egbert

**Affiliations:** 1 Department of Oral and Maxillofacial Surgery, University of Washington, 4800 Sand Point Way NE, Seattle, WA 98105 USA; 2 Pediatric Rheumatology, Mayo Clinic, Rochester, MN USA; 3 Pediatric Rheumatology, Seattle Children’s Hospital, University of Washington, Seattle, WA USA; 4 Pediatric Oral and Maxillofacial Surgery, Seattle Children’s Hospital, University of Washington, Seattle, WA USA

**Keywords:** Chronic nonbacterial osteomyelitis, Chronic recurrent multifocal osteomyelitis, Mandible, Treatment, Biopsy

## Abstract

**Background:**

Studies evaluating treatment responses for chronic nonbacterial osteomyelitis (CNO) are lacking. We aimed to measure and compare response rates of medical treatments, time to response of medical treatments among patients with CNO of the mandible, and describe bacterial contamination rates from biopsy.

**Methods:**

We conducted a retrospective chart review of all patients diagnosed with CNO of mandible between 2003 and 2017 and extracted demographic, clinical, laboratory, imaging and surgical data. Detailed medication use and response to medications were recorded. The primary outcome was response to medical treatments defined as improvement of presenting symptoms, inflammatory markers, and imaging if available. Medical treatments included nonsteroidal anti-inflammatory drugs (NSAIDs), glucocorticoids, disease modifying anti rheumatic drugs (DMARDs), anti-tumor necrosis factor (TNF) therapy, and pamidronate. Descriptive analysis was performed when appropriate. Multivariable logistic regression and Kaplan-Meier curves were applied to compare the responses to medical treatments and time to full response.

**Results:**

We identified 22 patients with a median age of 11 and 36% were female. Four patients (18%) had multifocal bone lesions. CT findings (*n* = 21) showed lytic lesions (62%) and sclerosis (90%). MRI (*n* = 14) revealed hyperintensity within bone marrow (100%), soft tissue (71%) and bony expansion (71%). Non-antibiotic treatments including NSAIDs (*n* = 18), glucocorticoids (*n* = 10), DMARDs (*n* = 9), anti-TNF therapy (*n* = 5) and pamidronate (*n* = 6) were applied. Rates of full responses to anti-TNF therapy (60%) and pamidronate (67%) were higher than that to NSAIDs (11%) (*p* < 0.05). Patients receiving pamidronate responded more rapidly than those receiving anti-TNF therapy (median two vs 17 months, *p* = 0.01) when there was a full response. All had bone biopsies. Intraoral biopsy was performed in 12 of 13 operated in our center and the most common contaminants were *Neisseria spp* and *Streptococcus viridians*.

**Conclusion:**

Both anti-TNF and pamidronate appeared superior to NSAIDs alone in treating mandibular CNO. Patients receiving pamidronate responded faster than those receiving anti-TNF therapy.

## Background

Chronic nonbacterial osteomyelitis (CNO) is a poorly described clinical entity in the Oral and Maxillofacial Surgery (OMS) literature due to the relative rarity of the disorder and unstandardized nomenclature [1–3]. The head and neck literature has used various terminology including Garré osteomyelitis, diffuse sclerosing osteomyelitis, primary chronic osteomyelitis, juvenile mandibular chronic osteomyelitis, chronic recurrent multifocal osteomyelitis (CRMO), and chronic nonbacterial osteomyelitis (CNO) [1]. Unilateral lesion is most common whereas bilateral lesions raise the suspicion of cherubism. CRMO and CNO are the most commonly used terms in the literature, particularly when other areas of the skeletal system are involved. CRMO was first described by Giedion et al. in 1972 [4] and is more common in children than SAPHO syndrome (synovitis, acne, pustulosis, hyperostosis, and osteitis). SAPHO syndrome has been postulated to affect the older patient counterpart along a continuum of CNO, but the afore-mentioned terms are classically considered multifocal bone disorders [5]. The diagnosis of CRMO is made by exclusion of other diseases. Manson et al. proposed the following diagnostic criterion for CRMO: 1) presence of two or more radiographically confirmed bone lesions, 2) prolonged course at least 6 months of exacerbations and remissions, 3) radiographic and scintigraphy evidence of osteomyelitis, 4) a lack of response to antimicrobial therapy of at least 1 month, and 5) lack of identifiable cause [6]. Jansson et al. proposed a diagnostic criteria supports that this is a diagnosis of exclusion, and CRMO falls within the subset of chronic nonbacterial osteomyelitis (CNO) [7].

Here we referred to the entire spectrum of diseases as CNO. This diagnosis of exclusion has most recently been described in the OMS literature by Padwa et al., consisting of a 22-patient case series [1]. Their report established the difficulty in reaching a final diagnosis in these patients. Only after seeing a mean of four providers with a median duration of 17 months of symptoms was a diagnosis made. Mandibular biopsies present a unique challenge for a diagnosis that relies on osteitis with a sterile culture. Due to the high prevalence of oral flora, extraoral biopsy was recommended over intraoral approach during the diagnostic workup to avoid oral microbial contamination [1], though this was not the objective of their study.

Treatment of CNO of the mandible is not standardized and reported modalities include surgical resection, hyperbaric oxygen, antibiotics, glucocorticoids, targeted biologic therapy, and/or bisphosphonates administration, among others [1, 8–12]. Patients are often initially treated with antibiotics for presumed infectious osteomyelitis regardless of bone culture results. When the diagnosis of CNO is suspected, long-term antibiotics are not indicated and may delay effective treatments. Variation in treatment exists across and within centers due to the paucity of comparative effectiveness data for CNO in general and particularly for mandibular CNO.

The specific aims of this study were to measure and compare responses to medical treatments, time to response of medical treatments, and describe bacterial contamination rates from biopsy among patients with CNO of the mandible treated at Seattle Children’s Hospital (SCH).

## Methods

### Study design

The authors designed a retrospective cohort study. Subjects who presented to SCH for clinical evaluation and management of CNO of the mandible between April 17th, 2003 and April 25th, 2017 were identified using our electronic medical record. Inclusion criteria were: 1) documented diagnosis of chronic nonbacterial osteomyelitis of the mandible; 2) age at initial onset was less than 18 years; 3) patients must have been seen at SCH with at least one follow up visit. ICD-10 codes were utilized to identify CNO diagnosis, codes included: acute osteomyelitis (M86.10), chronic osteomyelitis (M86.60), site specific to mandible (M27.2, M27.7), multifocal (M86.30), multiple sites (M86.39), and non-suppurating osteomyelitis (M86.6X-). Since charts from 2003 to 2015 were also analyzed, ICD-9 codes of 730.1 and 730.2 were also used to identify eligible patients. We validated our cohort using a manual chart review in order to include all potential CNO cases.

### Data collection

#### Outcome variables

The primary outcome was response to non-antibiotic medical treatments including nonsteroidal anti-inflammatory drugs (NSAIDs), glucocorticoids (prednisone, prednisolone, and methylprednisolone), methotrexate (MTX), sulfasalazine (SSZ), leflunomide (LEF), pamidronate, anti-tumor necrosis factor (TNF) therapy such as etanercept, adalimumab or infliximab. “No response” was defined as persistent pain, swelling and persistently elevated inflammatory markers, erythrocyte sedimentation rate (ESR) and C-reactive protein (CRP), and persistent abnormal signal on MRI, when available. “Partial response” was defined as improvement without complete resolution of pain, swelling, ESR, CRP and MRI imaging (when available). Some features may remain unchanged while all other features improved. “Full response” as well as “inactive disease” defined as resolution of pain, swelling, normalization of ESR, CRP and resolution/minimal abnormal signal on MRI. Because patients were treated with various sequences of non-antibiotics, non-NSAIDs, disease status at the initiation of a medication and at the subsequent visits while on same medication was recorded for Kaplan Meier curve analysis. When medications were used concurrently, mostly NSAIDs with pamidronate, anti-TNF therapy, glucocorticoids, MTX, SSZ, or LEF, the effects of the combined treatment was attributed to non-NSAIDs due to prior poor NSAIDs response in these patients.

#### Other variables

We extracted demographic and clinical data to identify predictors of response to treatment. Variables included age at diagnosis, follow up duration, CNO lesion distribution, detailed medication history, imaging findings from CT, bone scintigraphy and MRI, surgical procedures including biopsy approaches, microbiology report and laboratory findings. MRI of face or head without contrast comprising T1, short tau inverse recovery (STIR) from axial and coronal planes were obtained. Whole-body MRI was performed in one subject only because it was not adopted until 2017. Intraoral biopsy approach was defined by intraoral mandibular vestibular access described in the operatory note. Extraoral biopsy was defined by the classic Risdon technique^14^ described in the operatory note. Microbiology results of bone and tissue samples were reported separately when available.

### Statistical analysis

Descriptive analyses were performed when appropriate. Univariable logistic regression was performed to identify predictive covariates for response to each treatment. Multivariable logistic regression analysis was then performed to identify predictive covariates for response to each treatment while controlling for follow-up time. Additional multivariable logistic regression models controlling for follow-up time and and treatment duration were utilized. A Kaplan-Meier model and corresponding log-rank test were used to compare time to treatment response for those with full response within anti-TNF and pamidronate groups. We utilized a two-sided test of hypothesis for regression models. A *p*-value of < 0.05 was considered statistically significant for all analysis performed. Analyses were performed using Stata 14 (Stata Corp, College Station, TX).

## Results

A total of 22 patients were included in this study. Demographic and clinical characteristics are summarized in Table 1. The median age was 11 years old and eight (36%) of patients were female. The majority of patients, 15 (68%), were treated jointly between oral and maxillofacial surgery (OMS) and pediatric rheumatology (PR) and all patients received a bone biopsy. Mandibular lesions were most often isolated, but four (18%) had lesions in other bones. Approximately 1/3 of patients saw an outside OMS before being referred to SCH. Nine patients (41%) received biopsies before evaluation at SCH, one patient received a resection (5%), and 16 patients (73%) received antibiotics before referral to SCH. At SCH, most biopsies were performed by intraoral approach (92%), and one child had an extra-oral biopsy. Eighteen (82%) were treated with NSAIDs at SCH, nine children (41%) received antibiotics at SCH.

**Table 1:** Demographic info and baseline characteristics at initial clinical visit with OMS or PR at SCH (*n* = 22)

Study Variable	Median (IQR) or number (frequency)
Female, n (%)	8 (36)
Age (yr)	11 (7–12)
HLA-B27 positivity, n (%) (15 available)	1 (7)
ANA positivity, n (%) (10 available)	3 (30)
ESR (mm/hr) at initial visit, (*n* = 20)	16 (9–35)
CRP (mg/dL) at initial visit, (n = 20)	2.3 (0.8–1.9)
Treating Service, n (%)	
Rheum and OMS	15 (68)
OMS only	3 (14)
Rheum only	4 (18)
Multifocal lesions, n (%)	4 (18)
Clavicle	1 (5)
Lower extremity (femur, tibia/ fibula, foot)	3 (14)
Upper extremity (radius/ ulna, humerus)	1 (5)
Pre-SCH care, n (%)	
Outside OMS	7 (32)
Biopsies	9 (41)
Resection	1 (5)
Antibiotics	16 (73)
SCH surgical care, n (%)	
Biopsies (intraoral)	12 (55)
Biopsies (extraoral)	1 (5)
Return to OR/ additional surgery*	2 (9)
SCH medical care, n (%)	
Antibiotics	9 (41)
NSAIDs	18 (82)
Glucocorticoids	10 (45)
DMARDs (MTX, sulfasalazine)	9 (41)
Pamidronate	6 (27)
Anti-TNF therapy	5 (23)

HLA: human leucocyte antigen; ANA: antinuclear antibody; OMS: oral maxillary surgery; ESR: erythrocyte sedimentation rate; CRP: c reactive protein; NSAID: nonsteroidal anti inflammatory drug; DMARD: disease modifying anti rheumatic drug; MTX: methotrexate. TNF: tumor necrosis factor;

*Return to OR/ additional surgery was for mandibular recontouring. Additional extractions/dento-alveolar procedures were excluded

### Imaging results

**Fig. 1:**
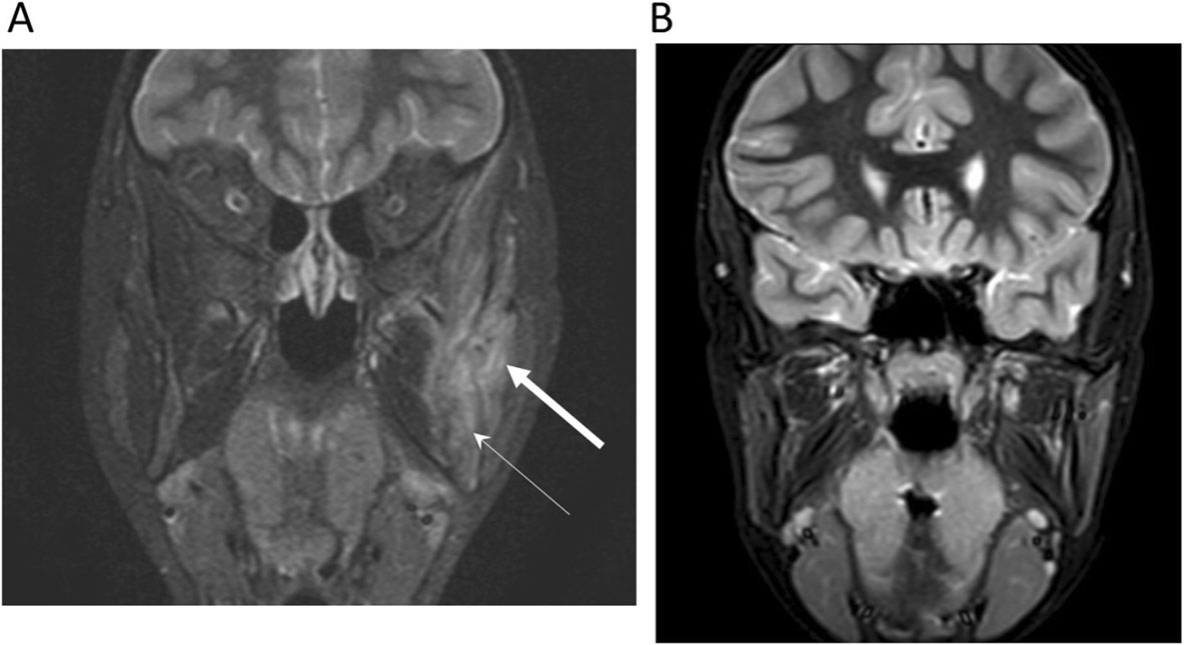
Representative MRI before (**a**) and after (**b**) infliximab (anti-TNF therapy). Thick arrow indicates soft tissue inflammation and thin arrow indicates hyperintensity within bone marrow and bony expansion of mandible. Soft tissue inflammation resolved and the abnormal hyperintensity within bone marrow and bony expansion resolved after treatment

Imaging findings showed common features including lytic lesions (62%) and sclerotic lesions (90%) on CT, increased uptake (100%) at affected area in bone scintigraphy and increased hyperintensity in bone marrow (100%), soft tissue (71%) as well as bony expansion (71%) in MRI. Only 4 patients (18%) had CNO lesions other than mandible. Subsequent MRIs were done on eight patients with six patients (75%) showing resolution or improvement of hyperintensity in bone marrow (Fig. 1, Table 2).

**Table 2:** Comparison of full response rates among different treatments

Medical Treatment at SCH	No Response	Partial response	Full Response	Odds ratio compared to NSAIDs group	95% confidence interval	*P* value
NSAIDs only (n = 18)	6	10	2	N/A	N/A	N/A
DMARDs (n = 9)	1	7	1	1	0.1–12.8	0.75
Glucocorticoids (n = 10)	0	10	0	N/A	N/A	N/A
Anti-TNF (n = 5)	0	2	3	12	1.2–121.6	<.05
Pamidronate (n = 6)	0	2	4	16	1.7–151.1	<.05

NSAID: nonsteroidal anti-inflammatory drug; DMARD: disease modifying anti rheumatic drug; N/A: not applicable

#### Outcomes after treatments

**Fig. 2:**
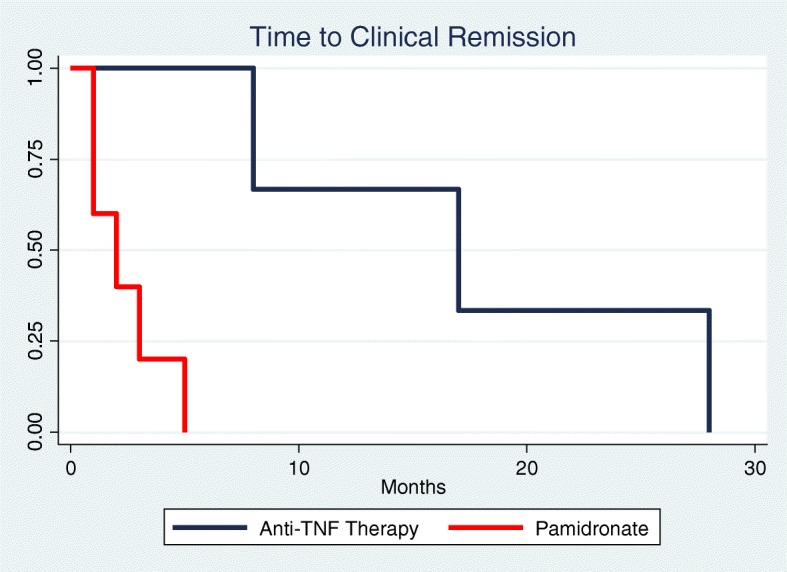
Time to full response in patients treated with pamidronate (*n* = 4) or anti-TNF therapy (*n* = 3) (*p* < 0.05)

A Kaplan Maier curve and corresponding log-rank test was performed to determine time to full treatment response. We only included those who had a full treatment response and whom had failed NSAID therapy to limit bias. Due to the low number of full respondents (0–2) in the other arms, only anti-TNF and pamidronate groups were compared. Time to full response was significantly shorter in pamidronate treatment group than anti-TNF treatment group (*p* < 0.05) as shown in Fig. 2.

We compared full response rate between NSAID only exposure group to other medical treatments (Table 3). The odds ratios compared to NSAIDs were one (*p* = 0.75), 12 (*p* < 0.05), and 16 (p < 0.05) for DMARDs, anti-TNF therapy, and pamidronate respectively and was not calculable for glucocorticoids. Some patients received various medications throughout the course. Detailed treatment history and responses were included in Table 4. A logistic regression model was created first in univariable and then multivariable stepwise fashion to identify predictors for any response or full response to the treatment groups. When controlling for follow-up time, treatment response was not associated with age, gender, or disease duration for any of the treatment arms. There again was a trend towards significance for younger patients being more likely to have any response to pamidronate (*p* = 0.059) while controlling for follow-up, this was not seen when looking at full response to pamidronate. We also performed the multivariable model accounting for antibiotic exposure at our institution prior to CNO diagnosis. There was no difference in treatment response between the antibiotics-exposed and antibiotics-unexposed patients.

**Table 3:** Initial imaging findings from MRI, bone scintigraphy, and CT and follow-up MRI findings^a^

	Number (frequency, %)
CT findings (n = 21)	
Lytic lesions	13 (62)
Sclerosis	19 (90)
Bone scintigraphy findings (*n* = 8)	
Increased uptake	8 (100)
MRI findings (n = 14)	
Hyperintensity in bone marrow	14 (100)
Hyperintensity in soft tissue	10 (71)
Bony expansion	10 (71)
Follow up changes	No change in 2 (25), decreased marrow hyperintensity in 6 (75)

^a^ One patient had bilateral lesions

**Table 4:** Demographics and Treatment

Subject	Sex	Age (yrs)	Disease duration at first visit (m)	Year of first visit	Follow up duration (m)	Disease status at last visit	Medication at last visit	Summary of disease course
1	M	11	24	2017	12	inactive	yes	Treated with 11 weeks of antibiotics and partially responded. Then treated with monthly pamidronate for 4 doses with partial response. Then switched to anti TNF therapy with full response.
2	F	15	11	2016	16	inactive	yes	Treated with 4 weeks of antibiotics and partially responded. Then treated with NSAIDS for 24 months with full response.
3	M	6	18	2012	58	inactive	yes	Treated with unknown duration of antibiotics with no response. Then treated with NSAIDS for 5 years with no response. MTX was also tried for 5 years and showed partial response. Responded partially from 3 days of steroid. Then switched to biologic for 3.5 years and showed partial response. Lastly switched to pamidronate with full response.
5	M	11	23	2012	8	inactive	yes	Treated with 12 weeks of antibiotics with no response. Then treated with NSAIDS for 10 months with full response.
6	F	12	6	2015	1	active	no	Treated with antibiotics with no response.
9	M	6	6	2013	16	active	yes	Treated with NSAIDS for 14 months with no response. Then switched to MTX/SSZ.
10	F	5	7	2016	16	inactive	yes	Treated with 1 month of antibiotics with no response. Then treated with 16 months of NSAIDS. Also received 12 months of MTX/SSZ with no response. Received monthly pamidronate for 6 doses with partial response. Then switched to anti TNF therapy with full response.
11	M	15	19	2012	29	inactive	no	Treated with 4 years of NSAIDS and partially responded. Received steroids with partial response. Then switched to MTX/SSZ with full response.
12	M	12	13	2012	23	inactive	yes	Received anti TNF therapy for 3.5 years with partial response.
13	F	11	4	2017	2	active	yes	Treated with 2 weeks of antibiotics with no response. Then treated with MTX/SSZ for 1 month and NSAIDS for 2 months with partial response.
15	F	3	6	2016	19	inactive	no	Treated with 11 days of antibiotics. Then switched to 2 months of steroids and 5 months of MTX/SSZ with partial response. Finally treated with monthly pamidronate for 11 doses with full response.
16	M	12	1	2015	15	active	no	Treated with 2 weeks of antibiotics. Then switched to 15 months of NSAIDs with partial response.
18	M	11	12	2003	84	inactive	no	Treated with >2 weeks of antibiotics with no response. Then treated with 70 months of NSAIDS with partial response. Received 4.5 months of steroids with partial response.
20	M	11	10	2008	55	active	yes	Treated with 2 months of antibiotics with no response. Treated with NSAIDS for 66 months with partial response. Then treated with 5 months of MTX/SSZ and 4 months of steroids with partial response.
21	F	14	36	2009	16	active	yes	Treated with 9 months of antibiotics and partially responded. Then treated with 19 months of NSAIDs with no response. Then switched to MTX/SSZ for 20 months with partial response. Also received 17 months of steroids with partial response.
22	M	12	0	2014	47	inactive	no	Treated with 6 weeks of antibiotics with no response. Received 2 years of NSAIDs with no response. Then treated with 28 months of MTX/SSZ with partial response. Finally received monthly pamidronate for 4 doses with with full response.
23	F	8	13	2016	2	active	yes	Treated with 3 weeks of antibiotics with no response. Then treated with 3 months of NSAIDs with partial response.
26	F	9	31	2016	14	inactive	yes	Treated with 2 months of antibiotics with no response. Then received 20 months of NSAIDs and intermittent steroids with partial response. Then switched to anti TNF therapy with full response.
27	M	11	42	2005	49	inactive	no	Treated with 3 weeks of antibiotics with no response. Then treated with 38 months of NSAIDs and 10 weeks of steroids with partial response.
29	M	7	36	2016	18	inactive	yes	Treated with 14 months of NSAIDs with no response. Then treated with 17 months of MTX/SSZ and 8 weeks of steroids with partial response. Switched to monthly pamidronate for 11 doses with full response.
30	M	7	4	2005	97	active	yes	Treated with 21 days of antibiotics with no response. Then treated with 8 years of NSAIDs with partial response.
31	M	11	several years	2008	90	active	yes	Treated with 96 months of NSAIDs and 55 days of steroids and partially responded.

*NSAID* nonsteroidal anti inflammatory durg, *MTX* methotrexate, *SSZ* sulfasalazine

### Microbiology results

Among our 22 patients, nine patients had pre-existing biopsies from outside providers, 13 patients received biopsies at SCH. One patient was biopsied by transcervical approach, which had no bacterial growth. The remaining 12 bone biopsies were performed by intra-oral approach. Two of these 12 patients had a mucosal biopsy adjacent to the bone sample as a reference sample to determine the oral flora. There were no apparent differences in microbial contaminants between mucosal and bone groups. Two of the intraoral bone biopsies were sterile (Table 5**)**. Otherwise, the most frequent contaminant was *Neisseria spp.*, followed by *Strep viridians* which are not pathogens for Chronic Osteomyelitis.

**Table 5:** Microbiology contaminants in bone cultures, all intraoral approach at SCH*

Flora	Patients, n (%)
Neisseria spp	6 (50)
Strep viridians	6 (50)
Haemophilus	5 (42)
Lactobacillus	1 (8)
Actinomyces	4 (33)
Rothia	2 (17)
Gemella	1 (8)
Kingella	1 (8)

* 9 patients had pre-existing biopsies from outside providers. 13 patients received biopsies at SCH, and one patient was by transcervical approach. All biopsies were bone biopsies. Two of the 12 intraoral bone biopsies had separate mucosal biopsies. Two of the intraoral bone biopsy group grew no organisms

## Discussion

We present a robust cohort of childhood onset mandibular CNO. We showed that the majority of patients were treated with NSAIDs. However, rates of full responses to anti-TNF therapy and pamidronate were significantly higher than that to NSAIDs monotherapy. The response to pamidronate had a trend towards occurring more often in younger patients and occurred faster than patients treated with anti-TNF therapy.

CNO was only first described in oral surgical literature in 1994 [2]. Delay and erroneous diagnosis of CNO may occur due to lack of knowledge or low clinical suspicion. The differential diagnosis of mandibular CNO includes infectious osteomyelitis, neoplasia (Ewing’s sarcoma and osteosarcoma), lymphoma, and Langerhan’s cell histiocytosis [1, 13]. The average patient age previously reported is 10 years, similar to our patient population [13]. Clinical symptoms including mandibular pain and swelling are common in CNO and was noted in our study. As previously reported, our cohort had a low prevalence of positive HLA-B27 or positive ANA. In Padwa’s study, trismus was found in 45% of patients, headache (18%), otalgia (18%), fever (9%), and exacerbation at night (23%) or during stress (27%) [1]. The numerous clinical symptoms and lack of laboratory abnormalities make diagnosis and monitoring of disease activity challenging. A sensitive or specific biomarker is not currently available.

Various radiographic studies have been described. Computed tomography of mandible shows findings similar to osteomyelitis, with initial stages showing bony lytic lesions and variable sclerosis, and later stages with bony expansion and periosteal thickening, without discrete abscess formation [14, 15]. Most of our patients have had CT and > 90% had signs of sclerosis and 62% with lytic lesions. In respect to radionuclide scans, mapping active foci by early uptake suggests inflammation with later uptake suggesting sclerosis, although the exposure from successive imaging is not desirable [13]. Eight of our patients received a bone scan and all had shown increased uptake. Only 18% of our subjects had imaging-detected non-mandibular lesions comparing to 68% reported by Padwa [1], which is likely due to the lack of consistent use of whole-body imaging. As shown in Padwa’s series, MRI provided the most comprehensive imaging assessment of CNO including mandibular contour alteration, high signal intensity with swollen masticatory muscles, periosteal hyperintensity, and sclerosis on pulse sequences [1]. In our study, 14 out of the 22 patients had at least one MRI and eight of those patients had at least one repeat MRI. All 14 patients had abnormal signal within bone marrow corresponding to active disease at initial evaluation. Bony expansion and abnormal signal within surrounding soft tissue were common. Abnormal signal within bone marrow was decreased in six of eight patients and these results suggested that MRI as a non-radiating imaging modality be considered as superior monitoring tool to other imaging modalities. While imaging is often used to monitor disease activity, standardized objective scoring systems are also lacking.

One of our study’s aims was to describe the bacterial contamination from various biopsy techniques for CNO. There is debate whether the aseptic extraoral approach outweighs the scarring it causes and the risk of damage to the facial nerve. Prior OMS literature reports the majority of biopsies to be from extraoral approach, but our study only biopsied one child by extraoral approach [1]. Given the small sample size, we were unable to statistically evaluate differences between the two groups. With that said, there was no change in medical management between children biopsied with either approach since both groups fit the CNO clinical and radiographic diagnosis. It remains unclear if bone biopsy is even necessary to diagnose CNO. Jansson et al. developed a score to diagnose CRMO without the need for bone biopsy [16]. They performed a retrospective cohort of 224 patients (102 with CRMO and 122 with other similar diseases), weighted variables (radiographic abnormalities and symptoms) ranging from 0 to 63, and applied the score to their diagnostic algorithm, but the validity remains to be confirmed in larger cohorts and prospective studies [16]. Our study of 22 children with CNO reported 10 out of 13 biopsies (12 intraoral) at SCH grew organisms. Contaminations were common which often led to a short course of antibiotics. However, prolonged antibiotics should be avoided.

The impact of antibiotics on CNO patients prior to appropriate therapy remains unfounded because we reported a high response rate (partial and full) to NSAIDs, DMARDs, glucocorticoids, biologic and pamidronate regardless of prior antibiotic exposure. In clinical practice, infection remains a valid concern at the beginning of diagnostic workup. When a patient fails to respond to appropriate antibiotics for likely organisms, CNO should be considered and further appropriate treatment may be initiated. As in our cohort, most patients received antibiotics for mandibular osteomyelitis as presumed infectious cause during their initial disease course. However, repeated antibiotics were given in some despite the lack of efficacy of antibiotics, which may have delayed definitive treatment. We felt that it was important to control for antibiotic exposure because CNO is a diagnosis of exclusion.

Treatment of CNO of the mandible is medical, and NSAIDs are generally accepted as a first-line treatment [15, 17, 18]. 18 of our 22 patients received NSAIDs during their treatment. More than half of them had some response, but only two (9%) had a full response. Glucocorticoid use is commonly reported in CNO cohorts, but the side effects of long-term glucocorticoid administration in this young patient population make this option less desirable [13, 19]. Ten (45%) of our patients received steroids, primarily during disease aggravation. Therefore, we were not surprised to see high rates of utilization of steroid sparing medical therapies. Among treatments, anti-TNF and pamidronate had significantly higher response rates than NSAIDs and DMARDs, which are similar to previous reports for CNO [20, 21]. When comparing patients who have received antibiotics at SCH with those who have not, we did not observe a significant difference in response to anti-TNF or pamidronate between two groups. The difference between two groups in their response to DMARDs was likely significant due to the low rate of complete response. In the recently published consensus treatment plans, DMARDs, anti-TNF and pamidronate were listed as equivalent choices [18]. Our data suggested that both pamidronate and anti-TNF therapy were better than NSAIDs. While we had a higher OR in the pamidronate group, 16 vs 12, our small sample size and wide confidence intervals preclude us from concluding that pamidronate is a more effective therapy. Time to event analysis showed a significantly shorter time to response in patients receiving pamidronate compared to anti-TNF therapy. Sample size should be considered while interpreting this data as well.

Other treatment modalities have been reported and include azithromycin, interferon, sulfasalazine, methotrexate, immunoglobulin, and colchicine [2, 13]. Only anti-TNF agents and pamidronate had significant higher rates to induce full response. Simm et al. described their experience with pamidronate in five pediatric patients failing NSAIDs (all lesions in the clavicle, hip, or leg, never in the mandible) and subjective symptoms were dramatically reduced in four out of five patients [22]. Hofmann et al. treated eight CNO children with pamidronate (1 mg/kg body weight) every 4 weeks for 6 months while continuing pre-existing NSAID therapy [23]. All patients showed at least some capacity of clinical remission of CNO during this period [23]. Six months after the last pamidronate dose, four patients showed complete clinical remission, three patients showed progression of CNO, and one patient was lost to follow-up [23]. Adverse effects of bisphosphonates in this patient population included nausea, phlebitis, headache, and fever [22, 23]. It is inconclusive whether there is increased risk of medication-related osteonecrosis of the jaws (MRONJ). Since 95% of those with MRONJ are elderly adults and most have underlying malignancy, such as multiple myeloma, it is felt that younger patients receiving bisphosphonates may be at decreased risk for developing MRONJ [24].

Our study has several limitations to consider. Firstly, data on NSAIDs may be biased because they are widely available over the counter. This may bias our estimates of disease duration as patients may have been treating or partially treating their disease prior to presentation. We accounted for this by only looking at NSAID failures for time to treatment response in our survival analysis. Secondly, our calculation or treatment response and time to response is affected by frequency of follow up. We did account for time based on documentation of when symptoms resolved as noted in patient records. Thirdly, our sample size may not have enough power to determine the statistically significant difference among treatment responses so this study needs to be repeated in a prospective larger-scale study. Lastly, there is no standardized score for determining treatment response. We utilized methods for determining this based on what is done in clinical practice when interpreting laboratory and imaging results in conjunction with clinical symptoms.

There are also important strengths in this study to consider. This is one of the largest cohorts of mandibular CNO reported to date, and the majority of our patients had received antibiotics without improvement prior to our evaluations. The collaboration between the OMS and rheumatology groups led to improved identification of CNO of mandible and the resulted in more timely and appropriate medical treatments.

## Conclusion

In conclusion, anti-TNF therapy or pamidronate appeared superior to NSAIDs at controlling CNO of mandible. Intraoral biopsy often yielded bacterial contamination, but helped to ruled out other potential etiologies in establishing an early diagnosis. Awareness of CNO as a source of jaw swelling and pain is important for oral surgeon community to understand. Collaboration with rheumatologists for assistance with medical management should be encouraged to improve outcomes and to allow for continued study and overall improvement in the management of this difficult disease.

## Data Availability

The data and materials used in this study can be made available on request.

## Abbreviations

ANA: Antinuclear antibodies
CNO: Chronic nonbacterial osteomyelitis
CRMO: Chronic recurrent multifocal osteomyelitis
CRP: C-reactive protein
CT: Computerized Tomography
DMARDs: Disease modifying anti rheumatic drugs
ESR: Erythrocyte sedimentation rate
HLA: Human leukocyte antigen
ICD-9/10: International classification of diseases
LEF: Leflunomide
MRI: Magnetic Resonance Imaging
MRONJ: Medication-related osteonecrosis of the jaws
MTX: Methotrexate
NSAIDs: Nonsteroidal anti-inflammatory drugs
OMS: Oral and maxillofacial surgery
PR: Pediatric Rheumatology
SAPHO: Synovitis, acne, pustulosis, hyperostosis, and osteitis
SCH: Seattle Children’s Hospital
SSZ: Sulfasalazine
TNF: Tumor necrosis factor
